# The prognostic value of BAP1, PBRM1, pS6, PTEN, TGase2, PD-L1, CA9, PSMA, and Ki-67 tissue markers in localized renal cell carcinoma: A retrospective study of tissue microarrays using immunohistochemistry

**DOI:** 10.1371/journal.pone.0179610

**Published:** 2017-06-27

**Authors:** Sung Han Kim, Weon Seo Park, Eun Young Park, Boram Park, Jungnam Joo, Jae Young Joung, Ho Kyung Seo, Kang Hyun Lee, Jinsoo Chung

**Affiliations:** 1 Department of Urology, Center for Prostate Cancer, Research Institute and Hospital of National Cancer Center, Goyang, Korea; 2 Department of Pathology, Center for Prostate Cancer, Research Institute and Hospital of National Cancer Center, Goyang, Korea; 3 Biometrics Research Branch, Division of Cancer Epidemiology and Prevention, Research Institute and Hospital of National Cancer Center, Goyang, Korea; Seoul National University College of Pharmacy, REPUBLIC OF KOREA

## Abstract

**Objective:**

To assess the prognostic roles of BAP1, PBRM1, pS6, PTEN, TGase2, PD-L1, CA9, PSMA, and Ki-67 tissue biomarkers in localized renal cell carcinoma (RCC).

**Methods:**

Patients who underwent a nephrectomy during 1992–2015 and had a primary specimen of their kidney tumor were included. The nine tissue biomarkers were immunohistochemically stained on tissue microarrays of RCC, and the semi-quantitative H-score, including intensity score, was used to grade the sample. The Cox proportional hazards model was used to evaluate tissue markers significant for overall survival (OS), cancer-specific survival (CSS), and recurrence-free survival (RFS) after adjusting for significant clinicopathological parameters.

**Results:**

Samples from 351 RCC patients were included. The mean age of the patients was 53.9 years; the rates of pathologic T1-2/≥T3 stage, Fuhrman 1+2/3+4 grade, recurrence, and death were 269/65(80.5/19.5%), 222/107 (67.5/32.5%), 6.6%, and 10.5%, respectively. Median OS, CSS, and RFS were 220.6, 220.6, and 147.1 months, respectively. The multivariable analysis showed that pathologic T stage and Fuhrman nuclear grade were significantly associated with OS and CSS. Pathologic T stage and tumor size were associated with RFS. After adjusting for these significant prognostic clinicopathological factors, Ki-67 was significantly associated with OS (hazard ratio [HR], 2.7), CSS (HR, 3.82), and RFS (HR, 4.85) and pS6 was associated with CSS (HR, 8.63) and RFS (HR, 8.51) in the multivariable model (p<0.05).

**Conclusion:**

pS6 and Ki-67 are significant prognostic factors of RCC; however, BAP1, PBRM1, TGase 2, PD-L1, CA9, PTEN loss, and PSMA markers did not show this association.

## Introduction

Two-thirds of all newly diagnosed renal cell carcinoma (RCC) cases are localized [1], with 30–40% of these cases progressing to metastatic disease, despite complete surgical resection. Overall, the morality rate for RCC is 20–40% [1–3]. This high mortality rate for RCC is due to a lack of confirmed, efficacious therapeutic options for long-term tumor control. The difficulty in developing new treatment for RCC lies in its resistance to radiotherapy, chemotherapy, and immunotherapy [4], intra- and intertumor heterogeneity, and the heterotypic characteristics of pleomorphic RCC histology [5, 6].

Prognostic markers of RCC are important for clinicians to prevent recurrence following surgical therapy. Significant prognostic clinicopathological features such as TNM stage, Fuhrman nuclear grade, histologic subtype, lymphovascular invasion and sarcomatoid differentiation have been identified [4]; however, these have shown limited prognostic value. No molecular biomarker has been identified in RCC thus far [4].

Tissue microarrays (TMA) combined with immunohistochemistry (IHC) permits the analysis of large cohorts, leading to a better understanding of disease pathogenesis, differentiating between disease characteristics, and determining the origin of metastatic cancers without a clear tissue of origin. It can encompass a wide spectrum of diverse tumor presentations and disease states for tumor marker analysis in order to predict clinical behavior and prognosis [7]. Herein, we conducted IHC of a TMA of RCC that was resected via radical or partial nephrectomy. Nine potential, tissue-based biomarkers were evaluated to ascertain their prognostic value in predicting recurrence and survival in a cohort of patients with localized RCC. The nine tissue markers selected relate to either the hypoxia inducible factor (HIF-1) pathway, or the phosphatidylinositol 3-kinase pathway. The HIF-1 pathway, consisting of von Hippel Lindau, HIF-1, and vascular epithelial growth factor, and the phosphatidylinositol 3-kinase pathway, which includes protein kinase B and mammalian target of rapamycin (PI3K/Akt/mTOR), are two important molecular pathways responsible for oncogenesis, disease progression, metastasis, and neovascularization in RCC [4, 6, 8]

## Materials and methods

### Ethics statement

Following approval of this retrospective study by the Institutional Review Board (IRB) of the National Cancer Center (IRB No. NCC 2015–0219), an exemption was granted for the need of written consent from patients. This study was conducted according to the principles expressed in the Declaration of Helsinki.

### Patients’ criteria and tissue samples

All patients with RCC who underwent either a radical or partial nephrectomy between 1992 and 2015, with available primary tumor and control specimens of the kidney cancer were included in the study. All samples were reviewed retrospectively by one 15-year experienced uropathologist (WSP) in a blinded manner, according to the guidelines of WHO/International Society of Urological Pathology (ISUP) consensus conference [9]. The medical records of the patients were obtained from a prospectively collected RCC registry database.

### IHC and assessment of the TMA

The IHC of the TMA was performed using a previously described method [10]. Thirty TMA blocks were built from representative tumor areas and paired, normal control tissue from formalin-fixed, paraffin embedded (FFPE) tumor material [11]. Duplicate cores, 2.0 mm in diameter, were taken from the tumor block and arrayed in recipient blocks to form the RCC TMA. Briefly, suitable areas for tissue retrieval were identified with standard hematoxylin/eosin stained sections, and all tissue was reviewed to confirm both the inclusion of appropriate tissue, as well as to ensure consistency in morphological assessment.

The nine markers, BRCA1 associated protein-1 (ubiquitin carboxy-terminal hydrolase) (BAP1), polybromo 1 of chromatin-histone regulator gene (PBRM1), phosphorylated S6 protein (pS6), phosphatase and tensin homolog (PTEN), tissue glutaminase, protein-glutamin γ–glutamyltransferase (TGase-2), programmed death-ligand 1 (PD-L1), carbonic anhydrase 9 (CA9), prostate-specific membrane antigen (PSMA), and Ki-67 were assessed by IHC staining of the TMA blocks, using a standard protocol and an automated immunostainer (Ventana, Benchmark, AZ, USA). After deparaffinization of the FFPE block, heat-induced antigen retrieval was performed in solution, according to the standard protocol (S1 Fig), and reactivity was measured using the Ultra-View detection kit (Ventana, Tucson, AZ, USA).

The expression score of IHC, defined as the staining intensity multiplied by the percent tumor positive area, was semi-quantitatively determined by the H-score (0–300), calculated by the multiplication of the intensity score (0–1, negative staining; 2–3, positive staining) by the area of expression (0–100%) [8, 12, 13]. A single uropathologist (WSP), blinded to the clinical outcome, assisted by one urologist (SHK), determined the H-score using the TMA. The loss of PTEN, BAP1, or PBRM1, were calculated as the inverse of the normal H-score.

### Statistical analysis

To examine the prognostic value of these tissue biomarkers in terms of overall survival (OS), cancer-specific survival (CSS), and recurrence-free survival (RFS), previously identified clinicopathological variables associated with prognosis were analyzed using a Cox proportional hazards model. Significant clinicopathological variables in the univariable model were included into a multivariable model of clinicopathological variables, using a backward variable selection method with an elimination criterion of 0.05. After adjusting significant clinicopathological variables, the nine tissue biomarkers were respectively evaluated in the final multivariable model. The results were presented as hazard ratios (HR) with their 95% confidence intervals (CI). P-values of less than 0.05 were considered statistically significant. All statistical analyses were performed using SAS version 9.3 (SAS Institute Inc., Cary, NC, USA).

## Results

The mean age of the patients was 53.9 years (males, 244 (69.5%]), and the rate of T1-2 pathological stage, Fuhrman 1+2/3+4 grade, recurrence, and death were 269 (80.5%), 222 (67.5%), 6.6% (n = 23), and 10.5% (n = 37), respectively. Median OS, CSS, and RFS were 220.6, 220.6, and 147.1 months, respectively. The remaining clinicopathological characteristics of the patients and the results of IHC staining for each tissue biomarker are summarized in Table 1.

**Table 1:** Baseline characteristics.

Variables	Mean±SD or N(%)
Age	53.85 ± 12.43
BMI	24.54 ± 3.46
Tumor size (mm)	45.55 ± 29.88
Gender (Male/Female)	244 / 107 (69.5 / 30.5)
Diabetes	44 (12.6)
Hypertension	114 (32.8)
Smoking	
no-smoker	152 (43.8)
ex-smoker	75 (21.6)
smoker	120 (34.6)
ASA 1/2+3	140 / 195 (41.8 / 58.2)
Stage T1-2/T3-4or N+	269 / 65 (80.5 / 19.5)
Fuhrman grade 1+2/3+4	222 / 107 (67.5 / 32.5)
Sarcomatoid differentiation	10 (2.9)
Necrosis	150 (44.0)
Lymphovascular invasion	31 (9.1)
Capsular invasion	33 (9.7)
No recurrence / Recurrence	328 / 23 (93.4 / 6.6)
Recurrence-free survival (median months, range)	147.1 (0.7–147.1)
Alive/Death	314 / 37 (89.5 / 10.5)
Overall survival (median months, range)	220.6 (1.6–220.6)
Cancer-specific survival (median months, range)	220.6 (1.6–220.6)
**Immunohistochemical markers (%)**	
BAP1(positive/loss)	288 / 63 (82.1 / 17.9)
PBRM1(positive/loss)	143 / 208 (40.7 / 59.3)
pS6(positive/negative)	15 / 336 (4.3 / 95.7)
PTEN(positive/loss)	223 / 128 (63.5 / 36.5)
TGase2(positive/negative)	205 / 146 (58.4 / 41.6)
PD-L1(positive/negative)	108 / 243 (30.8 / 69.2)
CA9(positive/negative) (miss = 1)	273 / 77 (78.0 / 22.0)
PSMA(positive/negative)	13 / 338 (3.7 / 96.3)
Ki67(positive/negative) (miss = 1)	158 / 192 (45.1 / 54.9)

BMI, body mass index; ASA, American Society of Anesthesiologists

According to the univariable analysis, the clinicopathological parameters associated with poorer prognosis in terms of OS were male sex (hazard ratio (HR] 2.87, 95% confidence interval (CI] 1.12–7.38), diabetes (HR 2.36, CI 1.11–5.02), smoker (HR 2.65, CI 1.23–5.70), stage ≥T3 (HR 11.91, CI 5.70–24.88), Fuhrman grade 3+4 (HR 5.65, CI 2.63–12.18), sarcomatoid differentiation (HR 10.59, CI 4.34–25.84), lymphovascular invasion (HR 4.41, CI 2.11–9.18), tumor size (HR 1.02, CI 1.01–1.03), and capsular invasion (HR 3.03, CI 1.31–7.00); in terms of CSS were diabetes (HR 2.76, CI 1.15–6.60), stage ≥T3 (HR 33.58, CI 10.02–112.56), tumor size (HR 1.03, CI 1.02–1.04), Fuhrman grade (HR 9.86, CI 3.35–29.02), sarcomatoid differentiation (HR 17.23, CI 6.68–44.46), lymphovascular invasion (HR 6.20, CI 2.74–14.04), and capsular invasion (HR 3.89, CI 1.53–9.92); and in terms of RFS were stage ≥T3 (HR 8.30, CI 3.24–21.25), tumor size (HR 1.02, CI 1.01–1.03), Fuhrman grade 3–4 (HR 2.86, CI 1.15–7.12), sarcomatoid differentiation (HR 10.96, CI 3.13–38.39), lymphovascular invasion (HR 3.87, CI 1.39–10.75), and capsular invasion (HR 3.38, CI 1.10–10.35), all with p-value less than 0.05 (Table 2). The nine tissue biomarkers did not demonstrate any significant prognostic value in terms of OS, CSS, and RFS when analyzed by H-scores. The intensity scores, however, being either positive or negative, were significantly useful in the uni- and multivariable analyses. The Ki-67 was a significant prognostic biomarker for OS (HR 3.88, CI 1.82–8.25), CSS (HR 6.64, CI 2.28–19.35), and RFS (HR 3.55, CI 1.39–9.10). BAP1 loss was associated with a poorer OS (HR 2.13, CI 1.06–4.26), and PSMA with a shorter RFS (HR 4.05, CI 1.19–13.83) (p<0.05, Table 2).

**Table 2:** Univariable Cox proportional hazard model of the clinicopathological parameters and tissue markers.

	Overall Survival		Cancer Specific Survival		Recurrence Free Survival	
Variables	HR (95% CI)	p value	HR (95% CI)	p value	HR (95% CI)	p value
	(N = 351, event = 37)		(N = 351, event = 26)		(N = 351,event = 23)	
Age	1.03 (1.00–1.05)	0.066	1.01 (0.98–1.04)	0.576	1.02 (0.99–1.06)	0.266
Gender						
Female	1 (ref)		1 (ref)		1 (ref)	
Male	2.87 (1.12–7.38)	**0.029**	3.34 (1.00–11.16)	0.050	2.80 (0.83–9.50)	0.098
BMI	0.95 (0.88–1.02)	0.168	0.97 (0.88–1.07)	0.552	0.95 (0.88–1.03)	0.248
Diabetes	2.36 (1.11–5.02)	**0.026**	2.76 (1.15–6.60)	**0.023**	1.84 (0.62–5.52)	0.275
Hypertension	1.03 (0.50–2.11)	0.931	0.77 (0.31–1.96)	0.588	0.58 (0.19–1.73)	0.326
Smoking						
no-smoker	1 (ref)	**(0.045)**	1 (ref)	(0.130)	1 (ref)	(0.361)
ex-smoker	1.91 (0.75–4.85)	0.175	2.09 (0.70–6.24)	0.188	1.49 (0.43–5.10)	0.529
smoker	2.65 (1.23–5.70)	**0.013**	2.56 (1.02–6.41)	0.046	1.99 (0.77–5.09)	0.154
ASA						
1	1 (ref)		1 (ref)		1 (ref)	
2+3	1.61 (0.76–3.39)	0.215	1.59 (0.65–3.90)	0.313	2.29 (0.74–7.15)	0.153
Stage						
T1-2	1 (ref)		1 (ref)		1 (ref)	
T3≤	11.91 (5.70–24.88)	**<.001**	33.58 (10.02–112.56)	**<.001**	8.30 (3.24–21.25)	**<.001**
Tumor size	1.02 (1.01–1.03)	**<.001**	1.03 (1.02–1.04)	**<.001**	1.02 (1.01–1.03)	**<.001**
Fuhrman grade						
1+2	1 (ref)		1 (ref)		1 (ref)	
3+4	5.65 (2.63–12.18)	**<.001**	9.86 (3.35–29.02)	**<.001**	2.86 (1.15–7.12)	**0.024**
Sarcomatoid differentiation	10.59 (4.34–25.84)	**<.001**	17.23 (6.68–44.46)	**<.001**	10.96 (3.13–38.39)	**<.001**
Necrosis	1.19 (0.61–2.35)	0.612	1.76 (0.79–3.91)	0.166	1.46 (0.59–3.61)	0.411
Lymphovascular invasion	4.41 (2.11–9.18)	**<.001**	6.20 (2.74–14.04)	**<.001**	3.87 (1.39–10.75)	**0.009**
Capsular invasion	3.03 (1.31–7.00)	**0.010**	3.89 (1.53–9.92)	**0.004**	3.38 (1.10–10.35)	**0.033**
**Tissue markers**						
BAP1 loss	2.13 (1.06–4.26)	**0.033**	2.00 (0.86–4.63)	0.108	1.53 (0.60–3.91)	0.380
PBRM1 loss	1.86 (0.90–3.87)	0.096	2.33 (0.93–5.85)	0.072	0.81 (0.35–1.91)	0.637
pS6	1.34 (0.32–5.57)	0.692	2.05 (0.48–8.75)	0.331	2.85 (0.66–12.42)	0.162
PTEN loss	1.12 (0.58–2.18)	0.731	1.44 (0.66–3.17)	0.360	1.30 (0.56–3.04)	0.543
TGase2	1.02 (0.52–1.97)	0.961	1.27 (0.56–2.89)	0.561	1.85 (0.72–4.74)	0.203
PD-L1	0.75 (0.35–1.60)	0.456	0.72 (0.29–1.80)	0.477	1.42 (0.60–3.37)	0.425
CA9	1.02 (0.47–2.25)	0.952	1.59 (0.54–4.65)	0.396	1.61 (0.54–4.85)	0.397
PSMA	2.02 (0.62–6.60)	0.242	1.95 (0.46–8.27)	0.365	4.05 (1.19–13.83)	**0.026**
Ki67	3.88 (1.82–8.25)	**<.001**	6.64 (2.28–19.35)	**<.001**	3.55 (1.39–9.10)	**0.008**

HR: hazard ratio, CI; confidence interval; BMI, body mass index; ASA, American Society of Anesthesiologists

**Fig 1:**
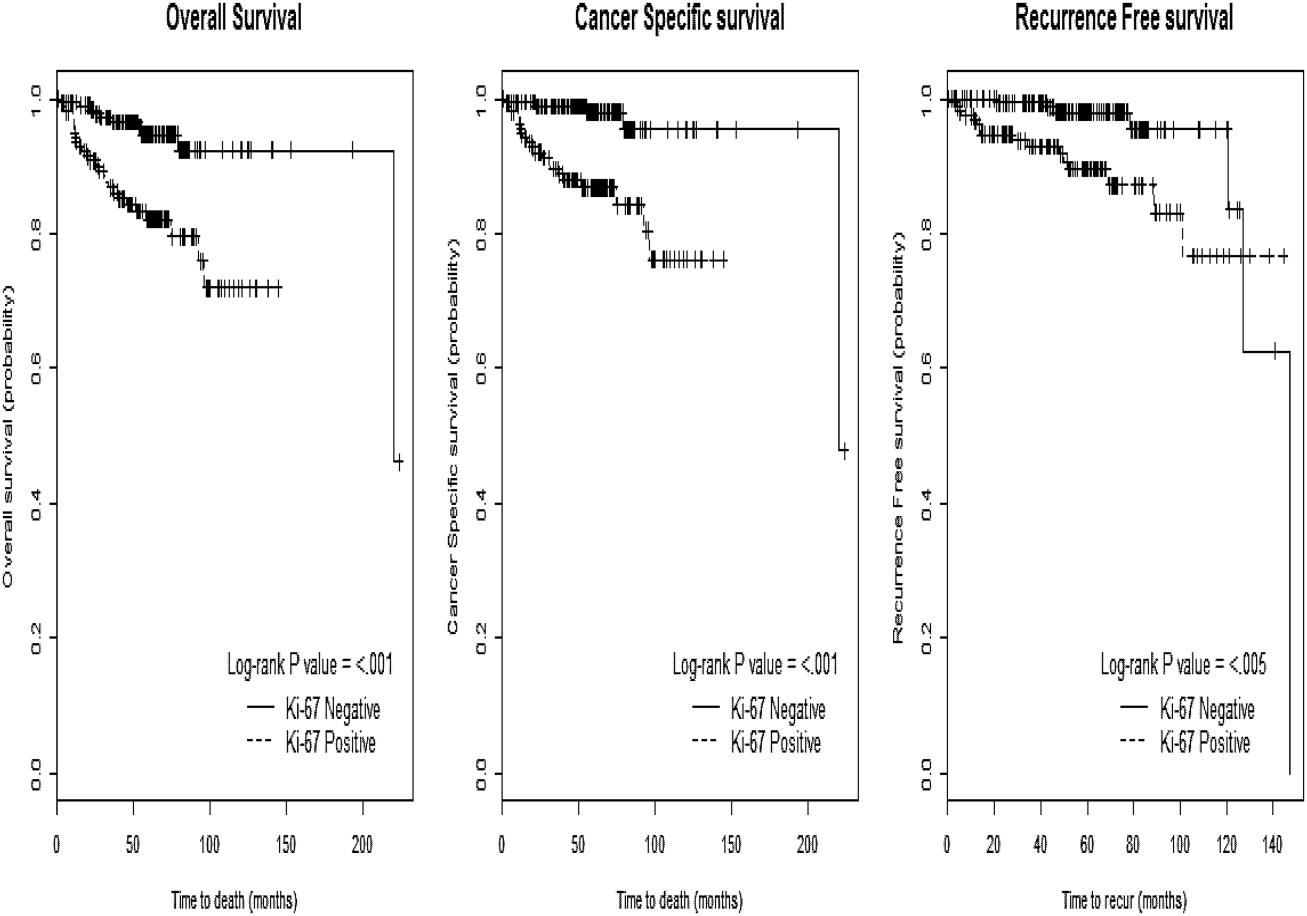
The Kaplan-Meier curves of Ki-67 according to (A) overall survival, (B) cancer-specific survival, and (C) recurrence-free survival.

**Fig 2:**
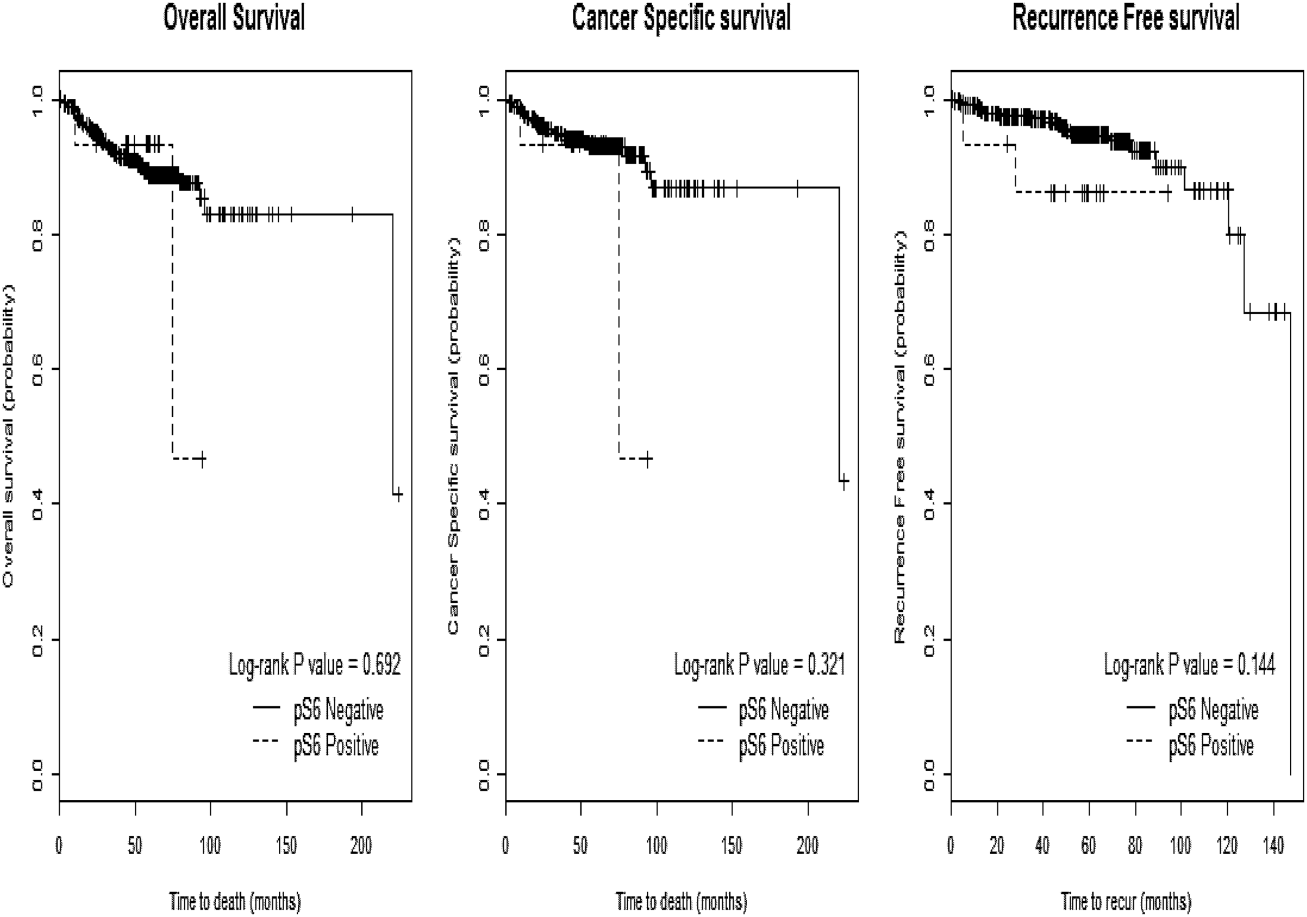
The Kaplan-Meier curves of pS6 according to (A) overall survival, (B) cancer-specific survival, and (C) recurrence-free survival.

In the multivariable analysis after adjusting for the significant prognostic clinicopathological variables identified above, pathologic T stage and Fuhrman grade were associated with poorer OS and CSS, and pathologic T stage and tumor size were associated with a shorter RFS (p<0.05). Ki-67 remained prognostic for OS (HR 2.70, CI 1.15–6.35), CSS (HR 3.82, CI 1.11–13.10), and RFS (HR 4.85, CI 1.39–16.96) (p<0.05, Fig 1), whereas BAP1 loss and PSMA did not demonstrate any significant prognostic value (p>0.05). Unlike the univariable results, pS6 was associated with a poorer CSS (HR 8.63, CI 1.78–41.91) and RFS (HR 8.51, CI 1.71–42.33) in multivariable model (p<0.05, Table 3) (Fig 2).

**Table 3:** Multivariable Cox proportional hazard model of tissue markers adjusted for significant prognostic clinicopathological variables/

	Overall Survival		Cancer Specific Survival		Recurrence Free Survival	
Marker	HR (95% CI)	p value	HR (95% CI)	p value	HR (95% CI)	p value
	(N = 351, event = 37)		(N = 351, event = 26)		(N = 351, event = 23)	
BAP1 loss	1.53 (0.75–3.16)	0.245	1.33 (0.56–3.16)	0.524	1.23 (0.45–3.39)	0.683
PBRM1 loss	1.02 (0.48–2.17)	0.958	1.09 (0.42–2.80)	0.862	0.64 (0.25–1.63)	0.344
pS6	3.53 (0.80–15.60)	0.096	8.63 (1.78–41.91)	**0.008**	8.51 (1.71–42.33)	**0.009**
PTEN loss	0.74 (0.37–1.47)	0.386	0.89 (0.39–2.03)	0.782	0.78 (0.30–2.00)	0.597
TGase2	1.43 (0.71–2.88)	0.323	1.68 (0.72–3.93)	0.232	2.68 (0.95–7.55)	0.062
PDL1	1.19 (0.55–2.59)	0.654	1.16 (0.46–2.98)	0.751	1.38 (0.52–3.68)	0.514
CA9	1.31 (0.56–3.06)	0.532	1.64 (0.55–4.87)	0.372	1.77 (0.53–5.97)	0.357
PSMA	1.30 (0.39–4.29)	0.667	1.16 (0.27–5.01)	0.839	3.35 (0.95–11.83)	0.060
Ki67	2.70 (1.15–6.35)	**0.023**	3.82 (1.11–13.10)	**0.033**	4.85 (1.39–16.96)	**0.013**

HR: hazard ratio, CI; confidence interval

## Discussion

For several decades, clinicians have observed the limited accuracy and value of the currently used clinicopathological variables and have attempted to search for new specific molecular tissue biomarkers [6, 14]. This study found pS6 for CSS and RFS, and Ki-67 for OS, CSS, and RFS, as significant prognostic markers for survival among the nine selected tissue markers closely related to RCC oncogenesis and tumor progression (p<0.05, Table 3).

The expression levels of each of the tissue markers were similar to those reported in previous studies, as shown in Table 1 [15, 16]. Expression of PSMA (3.7%) and pS6 (4.3%) were lower than that in previous studies [17, 18], while PTEN loss and Ki-67 expression were higher [15, 19] (Table 1). This difference in the expression rate of some tissue markers are related closely to differing baseline tumor tissue characteristics.

The pS6 protein was more frequently expressed in metastases than in the primary RCC [18]. Overexpression of pS6 has been associated with sensitivity to inhibitors of mTOR in previous studies, and are more likely to be expressed in metastases, suggestive of a poorer prognosis in RCC, in line with our findings (CSS with HR 8.63, CI 1.78–41.91, p = 0.008) (Table 3). The Ki-67 biomarker is well known as a marker of cellular proliferation, relating to neovascularization, proliferation, and progression in RCC [20, 21]. A significant association of Ki-67 expression with prognostic factors such as pathologic T stage, Fuhrman grade, and nodal and metastatic status has already been demonstrated [22]. This study also demonstrated its potential in predicting OS, CSS, and RFS in RCC, even after adjusting for significant prognostic clinicopathological parameters (HR >1.0, p<0.05, Table 3).

As for the other candidate tissue biomarkers, this study showed none to be significantly prognostic. The BAP1 loss and PBRM1 loss were recently found to be poor prognostic biomarkers for clear cell RCC, whereas Kapur et al. [23] demonstrated a more favorable prognosis with PBRM1 loss than BAP1 loss, and a significantly worse prognosis with combined BAP1 and PBRM1 loss. This study failed to show any significant prognostic role of BAP1 and PBRM1 loss after adjusting for the significant prognostic clinicopathological parameters, but their hazard ratios in uni- and multivariable analyses indicated poor prognostic value (HR > 1.0, p>0.05, Tables 2 and 3) without these adjustments (S1 Table). BAP1 and PBRM1 loss were significant factors of poor prognosis for OS (HR 2.25, p = 0.022) and for CSS (HR 2.82 p = 0.027), respectively. It is still debated whether PBRM1 loss is associated with either a favorable or unfavorable prognosis [22, 23]. However, this study showed that PBRM1 loss and BAP1 loss were poor prognostic factors for OS and CSS (HR >1.0, Table 2), and but did not add significantly to the prognostic ability of existing clinicopathological variables. In Joseph et al’s IHC study with BAP1 and PBRM1 expression in clear cell RCC, PBRM1 and BAP1 expression did not add independent prognostic information to the Mayo Clinic SSIGN (stage, size, grade and necrosis) score [24].

The study showed PTEN loss, CA9, TGase-2, and PD-L1 were not prognostic markers (p>0.05, Table 3). PTEN loss is an mTOR pathway-related biomarker, and similar to pS6, poor prognosis, previously described as BAP1 loss, may indirectly relate to the mTOR pathway [23, 25]. CA9 is known as a good prognostic marker relating to the HIF-1 pathway in clear cell RCC [12, 23, 25]. PSMA represents the disease state related to RCC staging and neovascularization due to the hematogenous invasion or spread of renal tumor cells [17, 26]. TGase–2 [14, 27] and PD-L1 [25] were also recently described as ineffective prognostic markers, similar to in this study, and are related to inflammatory reactions and disease progression such as metastatic invasion or therapeutic resistance [14, 27, 28]. (Table 3)

As for clinicopathological factors, male sex (only for OS), diabetes (only for OS and CSS), smoker (only for OS), stage ≥T3, tumor size, Fuhrman grade 3+4, sarcomatoid differentiation, lymphovascular invasion, and capsular invasion were associated with prognosis in terms of OS, CSS and RFS in the univariable model (p<0.05, Table 2). In the multivariable model, male sex, stage ≥T3, tumor size, Fuhrman grade, and sarcomatoid differentiation were related only to the survival (p<0.05, S1 Table) similar to that reported in previous studies [4, 29, 30].

The study had several limitations, including its retrospective design, and some technical errors in TMA preparation. The expression levels of nine proteins were evaluated by IHC staining, which can be variable in experimental setting, as well as in the interpretation of the scoring. Nevertheless, this study is the first to evaluate the clinical implications of these nine biomarkers relating to the pathophysiology of RCC, including disease progression, in a large TMA-IHC series. Finally, this study showed that none of the markers, except for pS6 and Ki-67, had predictable roles in disease prognosis for primary RCC. A further study assessing differences between these prognostic factors for metastatic lesions and primary tumors using tissue biomarkers, and associations with prognostic effect in metastatic RCCs would be needed.

## Conclusion

This TMA study showed only pS6 and Ki-67 biomarkers are prognostic for survival after adjusting for clinicopathological parameters, whereas BAP1 loss, PBRM1 loss, TGase 2, PD-L1, CA9, PTEN loss, and PSMA markers did not have any prognostic role in determining OS, CSS, and RFS.

## Supporting information

S1 Fig Distribution of the Ki-67 and pS6 markers in the primary renal cell carcinoma specimen; (1) Representative photomicrographs of Ki-67 immunohistochemistry (X70 for A, and X100 for B). All immunohistochemical staining was scored as either positive (scores of 2 and 3) or negative (scores of 0 and 1); (2) Representative photomicrographs of pS6 (X60) immunohistochemistry; A, intensity score 0; B, intensity score 1; C, intensity score 2; D, intensity score 3.
(DOCX)

S1 Table Multivariable Cox proportional hazard model with backward selection among only clinicopathological parameters and among tissue biomarkers. (DOCX)
