# Supplementary material for: The prognostic value of BAP1, PBRM1, pS6, PTEN, TGase2, PD-L1, CA9, PSMA, and Ki-67 tissue markers in localized renal cell carcinoma: A retrospective study of tissue microarrays using immunohistochemistry
Source: PLoS One. 2017 Jun 27;12(6):e0179610. doi: 10.1371/journal.pone.0179610 (PMC5487017; doi:10.1371/journal.pone.0179610)

**Supplementary Figure 1.** Distribution of the Ki-67 and pS6 markers in the primary renal cell carcinoma specimen; (1) Representative photomicrographs of Ki-67 immunohistochemistry (X70 for A, and X100 for B). All immunohistochemical staining was scored as either positive (scores of 2 and 3) or negative (scores of 0 and 1); (2) Representative photomicrographs of pS6 (X60) immunohistochemistry; A, intensity score 0; B, intensity score 1; C, intensity score 2; D, intensity score 3.

(1)


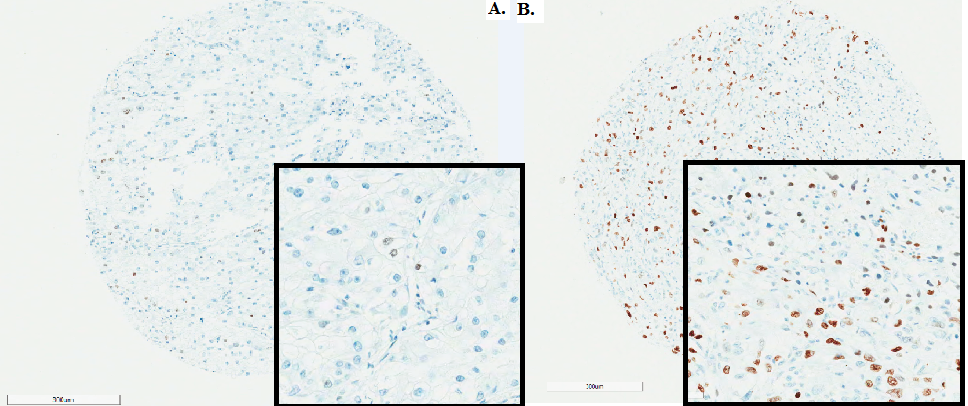


(2)


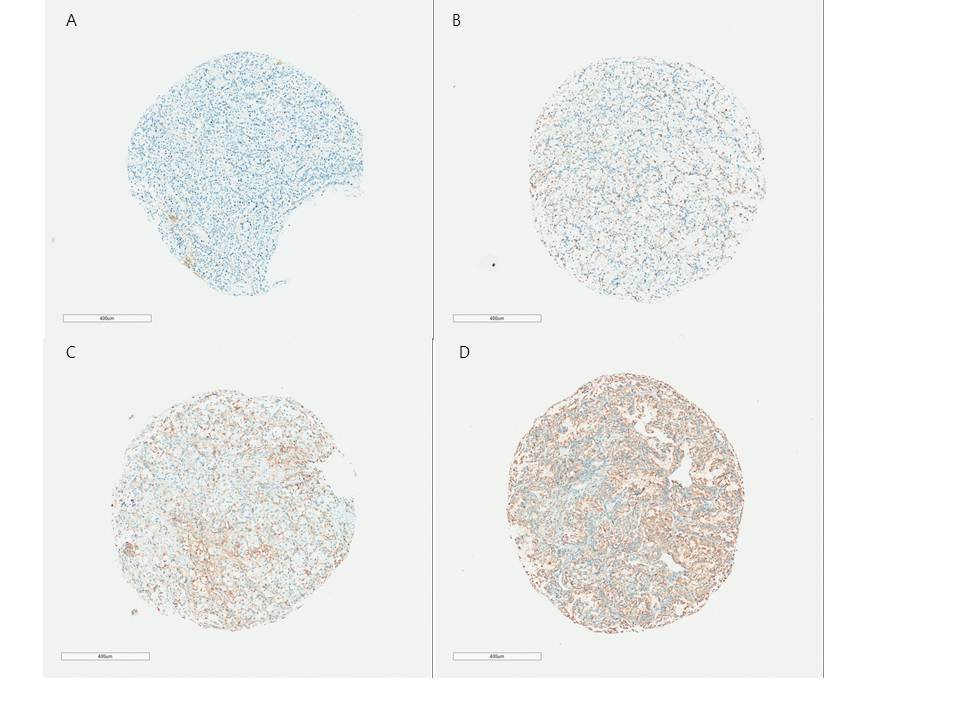

Supplement: S1 Fig — Distribution of the Ki-67 and pS6 markers in the primary renal cell carcinoma specimen; (1) Representative photomicrographs of Ki-67 immunohistochemistry (X70 for A, and X100 for B). All immunohistochemical staining was scored as either positive (scores of 2 and 3) or negative (scores of 0 and 1); (2) Representative photomicrographs of pS6 (X60) immunohistochemistry; A, intensity score 0; B, intensity score 1; C, intensity score 2; D, intensity score 3. (DOCX) [file pone.0179610.s001.docx]
