# Supplementary material for: The prognostic value of BAP1, PBRM1, pS6, PTEN, TGase2, PD-L1, CA9, PSMA, and Ki-67 tissue markers in localized renal cell carcinoma: A retrospective study of tissue microarrays using immunohistochemistry
Source: PLoS One. 2017 Jun 27;12(6):e0179610. doi: 10.1371/journal.pone.0179610 (PMC5487017; doi:10.1371/journal.pone.0179610)
Supplement: S1 Table — (DOCX) [file pone.0179610.s002.docx]

Supplementary Table 1. Multivariable Cox proportional hazard model with backward selection among only clinicopathological parameters and among tissue biomarkers.

|  | Overall Survival | | Cancer Specific Survival | | Recurrence Free Survival | |
| --- | --- | --- | --- | --- | --- | --- |
| **Variables** | HR (95% CI) | p value | HR (95% CI) | p value | HR (95% CI) | p value |
|  | (N=351,event=37) |  | (N=351, event=26) |  | (N=351,event=23) |  |
| Male Gender | 3.34 (1.01-11.08) | **0.048** |  |  |  |  |
| Stage ≥T3 | 7.19 (3.26-15.87) | **<.001** | 17.59 (5.02-61.61) | **<.001** | 3.98 (1.30-12.20) | **0.016** |
| Tumor size |  |  |  |  | 1.02 (1.00-1.03) | **0.011** |
| Fuhrman grade 3+4 | 2.80 (1.24-6.34) | **0.013** | 4.19 (1.38-12.71) | **0.011** |  |  |
| Sarcomatoid differentiation |  |  | 3.09 (1.01-9.44) | **0.048** |  |  |
|  | Overall Survival | | Cancer Specific Survival | | Recurrence Free Survival | |
| **Variables** | HR (95% CI) | p value | HR (95% CI) | p value | HR (95% CI) | p value |
| BAP1 loss | 2.25 (1.12-4.51) | **0.022** |  |  |  |  |
| PBRM1 loss |  |  | 2.82 (1.12-7.09) | **0.027** |  |  |
| Ki67 | 3.99 (1.87-8.50) | **<.001** | 7.41 (2.53-21.69) | **<.001** | 3.55 (1.39-9.10) | **0.008** |

HR: hazard ratio, CI; confidence interval
